# Transcriptional profiles of human islet and exocrine endothelial cells in subjects with or without impaired glucose metabolism

**DOI:** 10.1038/s41598-020-79313-y

**Published:** 2020-12-18

**Authors:** Alexander Jonsson, Anders Hedin, Malin Müller, Oskar Skog, Olle Korsgren

**Affiliations:** 1 Department of Immunology, Genetics and Pathology, Uppsala University, Uppsala, Sweden; 2 Institute of Medicine, University of Gothenburg, Gothenburg, Sweden

**Keywords:** Diabetes, Transcriptomics, Preclinical research, Cardiovascular biology

## Abstract

In experimental studies, pancreatic islet microvasculature is essential for islet endocrine function and mass, and islet vascular morphology is altered in diabetic subjects. Even so, almost no information is available concerning human islet microvascular endothelial cell (MVEC) physiology and gene expression. In this study, islets and exocrine pancreatic tissue were acquired from organ donors with normoglycemia or impaired glucose metabolism (IGM) immediately after islet isolation. Following single-cell dissociation, primary islet- and exocrine MVECs were obtained through fluorescence-activated cell sorting (FACS) and transcriptional profiles were generated using AmpliSeq. Multiple gene sets involved in general vascular development and extracellular matrix remodeling were enriched in islet MVEC. In exocrine MVEC samples, multiple enriched gene sets that relate to biosynthesis and biomolecule catabolism were found. No statistically significant enrichment was found in gene sets related to autophagy or endoplasmic reticulum (ER) stress. Although ample differences were found between islet- and exocrine tissue endothelial cells, no differences could be observed between normoglycemic donors and donors with IGM at gene or gene set level. Our data is consistent with active angiogenesis and vascular remodeling in human islets and support the notion of ongoing endocrine pancreas tissue repair and regeneration even in the adult human.

## Introduction

Endothelial cells are remarkably heterogeneous in different vascular beds^1^. Using > 32,000 endothelial cell single cell RNA-seq sets from 11 different murine tissues, Kalucka et al. constructed a murine endothelial cell transcriptome atlas containing 78 endothelial cell subclusters. They found that endothelial cells in mice differ between and within tissues and kinds of vascular beds, and that endothelial cell subsets in multiple tissues express markers associated with angiogenesis^2^.

Microenvironmental cues originating in the neighboring tissue are thought to be a co-regulator of endothelial cell heterogeneity, contributing to microvascular adaptations to local needs^3^. In the pancreas, β-cells and islet endothelial cells signal bidirectionally through soluble factors, extracellular matrix proteins, and cell-bound molecules^4^. β-cells secrete VEGF-A, which is seemingly an important factor for vessel size, density, and fenestration. Pancreatic endothelial cells have shown importance in β-cell differentiation during organogenesis^5^ and in stimulating the proliferation of β-cells^6,7^. Endothelial-to-β cell signaling facilitates glucose stimulated insulin secretion^8,9^ and increases islet insulin content^9^. Disrupted morphology of the islet microvasculature is correlated with reduced β-cell mass and diabetes in various rodent models^10^.

The endocrine pancreas is highly vascularized, with rat islets receiving roughly 10% of the pancreatic perfusion under basal conditions^11^ while only making up 1% of the pancreatic volume. Rat islet endothelium contains ten times as many fenestrae as the exocrine endothelium, with a sharp margin at the islet-exocrine interface^12^. In human organ donors without diabetes, islet microvascular vessels have a larger diameter and lower density than the exocrine microvasculature^13^. An increase in islet capillary density has been found in patients with type 2 diabetes compared with non-diabetic subjects^14^. Insulin has been shown to have general vasodilating effects, acting through local production of nitrous oxide in vascular endothelium^15^ among other mechanisms. A recent study^16^ found a general proliferation in all pancreatic cell types in organ donors with an increased duration of stay in the intensive care unit, suggesting that a general proliferation can be triggered in the human pancreas through hitherto unknown processes.

Thus, data from human and rodent studies suggest that islet endothelial cells have important roles in islet physiology. Based on the earlier findings of differences between microvascular beds, we expected to find differences between microvascular endothelial cells in the neighboring endocrine and exocrine compartments of the pancreas also at the gene expression level. Identifying such differences could grant insights into the endothelial characteristics necessary for maintaining normal function in the human pancreas. In this study we thus compared gene expression profiles of primary microvascular endothelial cells (MVECs) from human islets of Langerhans and exocrine tissue.

## Results

### Endothelial cells are highly enriched in sorted microvascular endothelial cell samples

**Figure 1:**
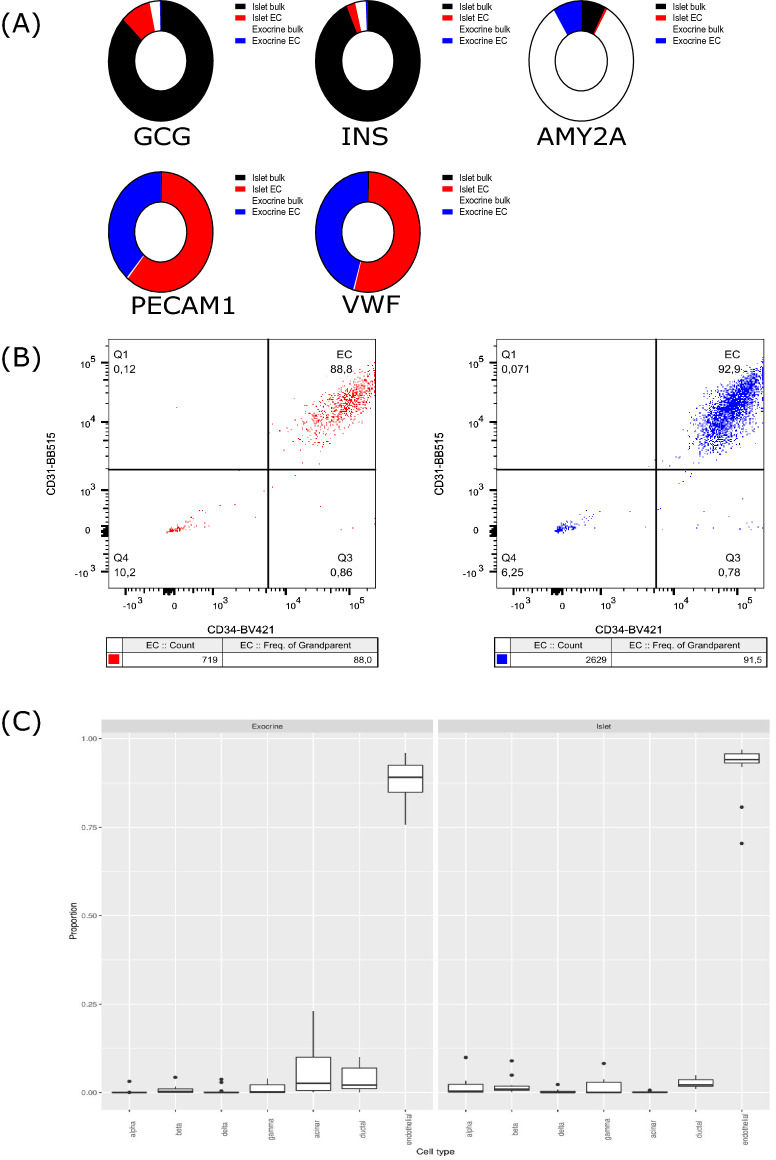
(**A**) Summarized RT-qPCR data for all included sorted samples and associated bulk samples. The sums of the 2^dCT values are used to plot the contribution of each sample type to the total expression of the target genes. Red—islet endothelial cell sample signal, blue—exocrine endothelial cell sample signal, black—bulk (non-sorted) islet tissue signal, white—bulk (non-sorted) exocrine tissue signal. n = 12 (islet bulk), 12 (islet EC), 13 (exocrine bulk) and 13 (exocrine EC). (**B**) Flow cytometric analysis of sorted cells. Dot plots are presented from one representative sorted islet sample (left—from normo2) and one representative sorted exocrine sample (right—from normo5), indicating that endothelial cells constitute 88% and 91.5% of all cells in these samples. All sorted samples were analyzed and samples closest to the median values were designated representative samples. (**C**) Deconvolution of RNA-Seq data into estimated proportions of alpha, β, delta, gamma, acinar, ductal, and endothelial cells in exocrine (left) and islet (right) tissue. The proportions were estimated from the raw counts using Multi-subject Single Cell Deconvolution (MuSic). All proportions sum to one. n = 13 (exocrine) and n = 12 (islets).

Relative expression levels of tissue-specific genes targeted by quantitative real-time PCR (RT-qPCR) are summarized in Fig. 1A. Endocrine and exocrine tissue genes were mainly detected in bulk samples from the corresponding tissue, with some signal also detected in the corresponding sorted samples. Endothelial genes *PECAM* and *VWF* were almost exclusively detected in sorted MVEC samples.

Using FACS, endothelial cell content was analyzed in aliquots collected from samples after sorting (Fig. 1B). After exclusion of debris, CD31^+^CD34^+^CD45^−^ (endothelial) cells constituted a median of 86% (range 66–92%) and 92% (range 56–98%) of events in sorted islet and exocrine samples respectively.

Through transcriptomics-based deconvolution of cellular constituents using a previously published data set^17^, a median of 92% (range 70–97%) of the cells in our sorted samples were identified as endothelial cells (Fig. 1C). FACS and transcriptomics-based deconvolution correlated well (data not shown).

### The tissue provenance of endothelial cells, but not glycemic status, is reflected in global gene expression

**Figure 2:**
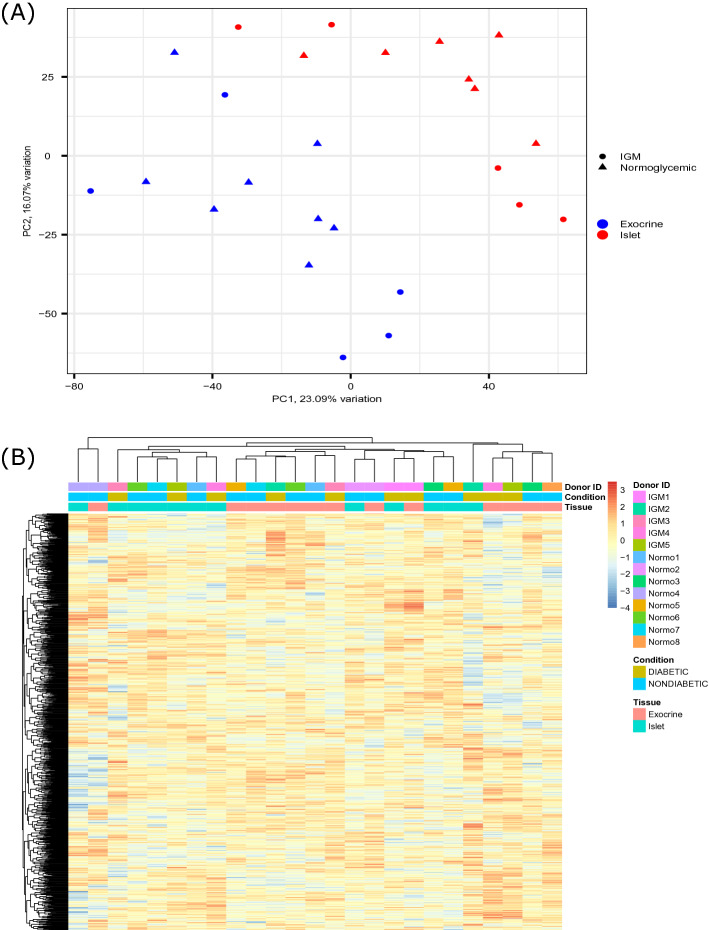
(**A**) Principal component analysis showing sample variation in two-dimensional principal component space for the sorted islet and exocrine endothelial cell samples. PC1 accounts for 23.09% of the total variation and PC2 16.07% of the total variation exocrine endothelial cell samples. PC1 accounts for 23.09% of the total variation and PC2 16.07% of the total variation. Circles indicate samples from IGM (Impaired Glucose Metabolism) donors, triangles indicate samples from normoglycemic donors. Red indicates islet MVEC samples, blue indicates exocrine MVEC samples. n = 12 (islets) and n = 13 (exocrine). (**B**) Heatmap with hierarchical clustering of genes and samples using the 1000 genes with the most variance. Color intensity indicates expression level, given as z-score of CPM- TMM- normalized counts. The colored bars on top indicates donor ID, condition (glycemic status) and tissue of origin respectively. A trend towards clustering by tissue of origin is evident. When samples from exocrine and islet cluster together, they originate from the same donor. n = 12 (islets) and 13 (exocrine).

Principal component analysis (PCA) revealed a trend towards islet- and exocrine endothelial cell samples forming two separate clusters (Fig. 2A). There was no obvious separation of normoglycemic donors and donors with IGM on PC1 or PC2. There was a slight tendency for samples from the same donor to associate (not shown).

For glycemic status, no differentially expressed genes or enriched gene sets were identified.

In a heatmap constructed using the 1000 genes with the most variance, islet and exocrine samples tended to create separate, somewhat overlapping clusters (Fig. 2B).

### Islet- and exocrine MVECs differ in expression of a large number of genes

**Figure 3:**
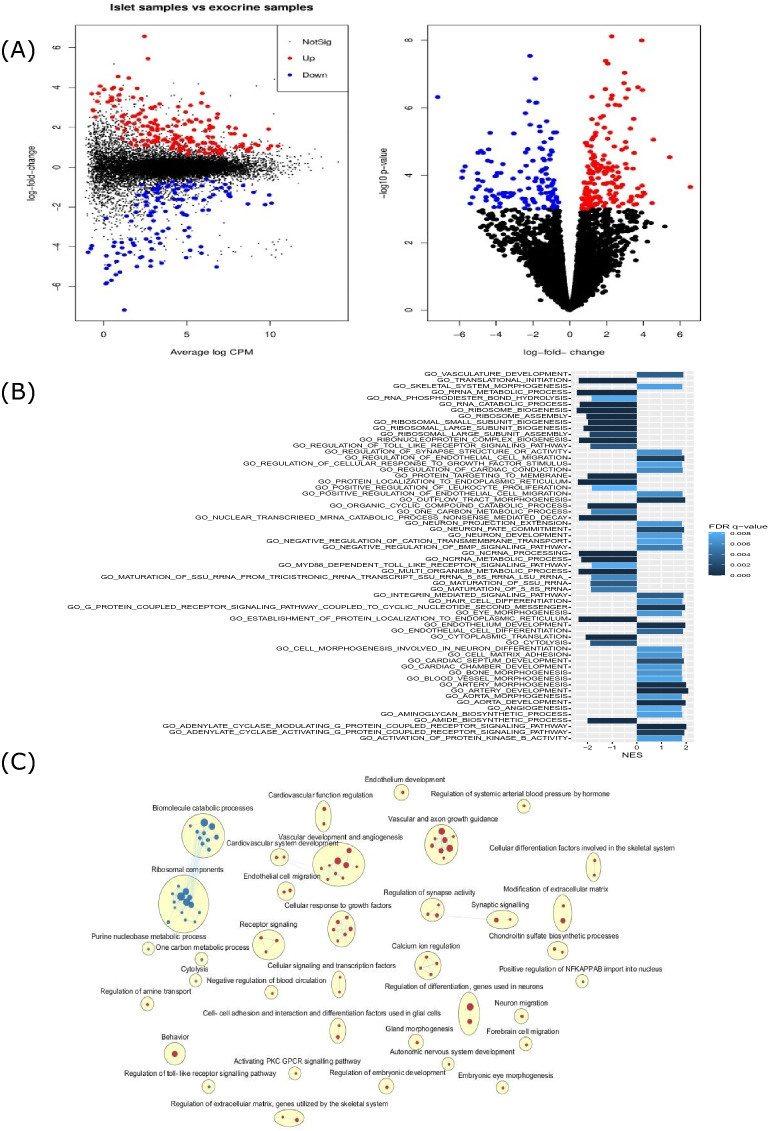
(**A**) Left: Mean difference plot comparing gene expression in islet- and exocrine MVEC samples, displaying log fold change and logCPM. Right: Volcano plot illustrating the logFC and log p-values of the same data, as estimated using a generalized linear model test for differential expression relative to a threshold (glmTREAT). Genes with a fold change of at least 1.2 at FDR < 0.05 are highlighted in red (indicating islet MVEC enrichment) or blue (indicating exocrine MVEC enrichment). n = 12 (islets) and 12 (exocrine). (**B**) The top enriched gene sets (FDR < 0.01) as determined with gene permutation pre-ranked GSEA, with the x-axis displaying normalized enrichment score (NES) and the color of the bar indicating the FDR q-value. A NES > 0 indicates enrichment in islet MVEC samples, a NES < 0 indicates enrichment in exocrine MVEC samples. n = 12 (islets) and 12 (exocrine). (**C**) Enrichment map showing relations between enriched gene sets (with FDR < 0.01 and nominal p-value < 0.005) as determined by gene permutation pre-ranked GSEA. Each gene set is represented by a node (red and blue dots for islet MVEC and exocrine MVEC enrichment respectively). Enriched gene sets have been hierarchically clustered, and most clusters (yellow ellipses) have been manually annotated. Edge widths indicate the degree of overlap between gene sets. The sizes of the circles are proportional to the number of genes in the gene set. n = 12 (islets) and 12 (exocrine).

A total of 13,451 genes passed the filtering (filterByExpr) step, and 1249 genes were found to be differentially expressed between islet and exocrine samples, using an FDR < 0.05 assuming a log fold change of ± log2 (1.2) as significance threshold. A total of 676 genes were enriched in islet samples, while 573 genes were enriched in exocrine samples (Fig. 3A). The full results of the differential gene expression analysis can be found in GEO (GSE157546).

Pre-ranked gene set enrichment analysis (GSEA) was performed using 2937 “gene ontology: biological processes” (GO:BP) gene sets containing 15–500 genes each. 347 gene sets were enriched in islet MVEC samples, and 138 gene sets were enriched in exocrine MVEC samples. The full GSEA results for all included gene sets are available online at GEO (GSE157546). The most enriched gene sets in islet samples contain several GO terms related to vascular development (Fig. 3B). The most enriched gene sets enriched in exocrine samples contain several GO terms related to ribosomal assembly and protein translation (Fig. 3B). Leading edge analysis was performed to identify gene sets with an overlap in the genes driving gene set enrichment. The overlap between enriched gene sets are visualized in Fig. 3C.

### Comparison to murine tissue-specific and vascular-process specific endothelial cell markers

**Figure 4:**
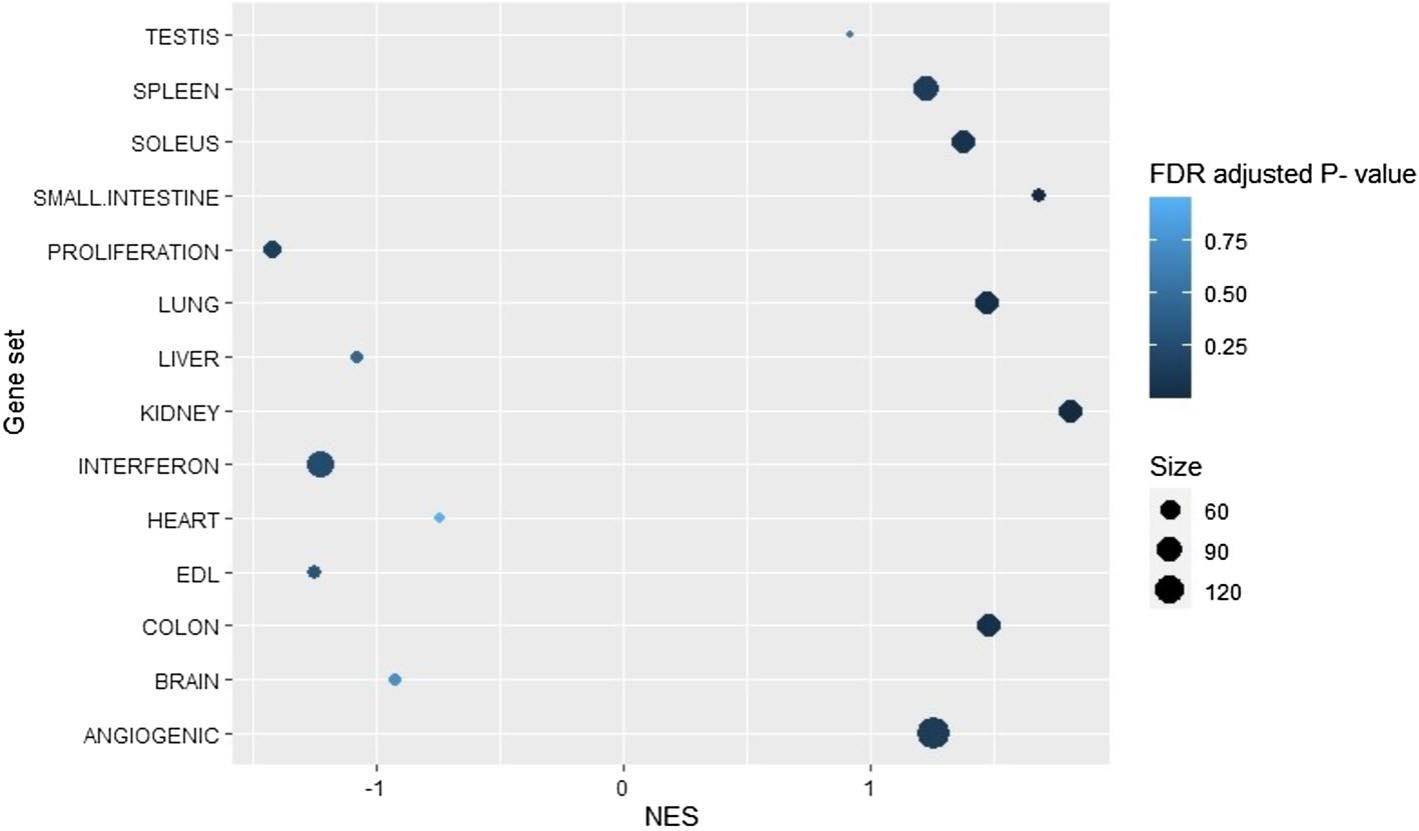
Results of the gene permutation pre-ranked gene set enrichment analysis (GSEA) using 14 capillary endothelial cell marker sets derived from the murine endothelial cell atlas. 11 of the sets are dependent on tissue of origin while 3 are biological processes. Normalized Enrichment Score (NES) is indicated by the x-axis. Positive NES values indicate enrichment in islet MVECs, negative NES values indicate enrichment in exocrine MVECs. FDR adjusted p-value is denoted by the color of the dots. The sizes of the gene sets are indicated by the size of the individual dots. n = 12 (islets) and 12 (exocrine).

Islet endothelial cell samples were enriched for kidney, small intestine, lung, colon, soleus and spleen (FDR < 0.25) marker sets (Fig. 4). The most enriched tissue set was “kidney” followed by “small intestine” and “colon”. No tissue set was significantly enriched in exocrine samples.

Islet endothelial cell samples were also enriched for the “angiogenic” marker set while the exocrine endothelial cell samples were enriched for the “proliferation” marker set (FDR < 0.25).

## Discussion

Transcriptional profiles of highly enriched human pancreatic MVECs from donors with normoglycemia and donors with IGM are presented. The most notable findings pertain to the identification of transcriptional differences between islet and exocrine MVECs. As determined by GSEA, multiple gene sets involved in general vascular development and extracellular matrix remodeling were enriched in islet endothelial cells.

Data from RT-qPCR, flow cytometry, and MuSic demonstrate that endothelial cells constituted an absolute majority of cells in the sorted samples. Tissue was processed immediately after islet isolation in order to minimize the impact of isolation- and culture associated changes to RNA expression. In conjunction with average logCPM being a factor in the significance testing, these quality control measures argue for the majority of the differences in gene expression reflecting true phenotypical differences between islet- and exocrine endothelial cells. It is important to note that there could exist subtypes of endothelial cells within the islets, and that the subtypes may have different transcriptional profiles. Notably, in the context of angiogenesis, endothelial tip cells would have a different transcriptional profile compared with other endothelial cells^2,18^. While single-cell RNA sequencing is a powerful tool, it is limited by a low fidelity of detection. Many genes found to be expressed by bulk RNA-sequencing cannot be reliably detected in any given cell using single-cell RNA sequencing^19^. To elucidate general differences between endothelial cells in islets and exocrine tissue we therefore instead opted for bulk RNA-seq.

Due to the paired nature of our islet- and exocrine MVEC samples, we performed pre-ranked GSEA for comparisons between tissues. Many of the enriched gene sets in islet EC compared with exocrine EC were related to angiogenesis and vascular development, endothelial cell development and migration, and cardiovascular system regulation. Several gene sets related to nerve development and differentiation were also found to be upregulated—this likely represents genes with dual functions. Endothelial tip cells and axonal growth cones share multiple features, with a large overlap in their signaling molecules. The gene sets related to axonal guidance could thus be enriched due to an upregulation of vascular guidance molecules. One of the most significantly upregulated genes in the islet samples was *UNC5B*, which is almost exclusively expressed in vasculature^20,21^, lending credence to the interpretation of genes with dual functions driving upregulation in nerve cell-related genes. Gene sets related to maintaining and modifying the extracellular matrix were upregulated in islet samples**,** supporting the notion of ongoing remodulation of the islet vasculature.

The findings at the level of gene sets are reflected by an upregulation of several genes that have previously been described to be involved in angiogenesis and morphological processes. PLVAP is known to be important for endothelial fenestration and has been suggested to be upregulated by VEGF-A^22^. Although mature vascular beds are thought to be largely quiescent and independent of VEGF-A signaling^23,24^, VEGF-A has been shown to influence vascular survival and fenestration in various endocrine organs in the adult mouse^23^. Both PLVAP and VEGF-A signaling appears to be upregulated in islets. This is consistent with a greater fenestration in human islet capillaries compared with exocrine capillaries. In mice, β-cells produce VEGF-A which is an important modulator of the islet vasculature and contributes to normoglycemia^6,14^. Notably, while inactivation of VEGF-A at any time point leads to reduced islet vascularization, inactivation during pancreatic development leads to reductions in β-cell mass and function^24^. Both VEGF-A and VEGF-C, as well as FLT1 and KLK, the genes encoding the VEGF receptors, were upregulated in islets in our study. This is consistent with a similar mechanism in humans as that previously described in mice. VEGF-A is an important mediator in angiogenesis, and the VEGF-A receptor FLT1 is typically upregulated in sprouting angiogenesis^21^.

It has previously been hypothesized that islets have a natural progression where they grow until the metabolic demand exceeds the vascular supply and a vascular catastrophe occurs^25^. The results presented herein are consistent with this hypothesis. The increased levels of vascular guidance molecules, such as VEGF-A and other markers of angiogenesis, is consistent with a higher level of angiogenesis in islets compared with exocrine tissue. If islets exist in a state of perpetual growth, a high degree of angiogenesis and vascular remodeling would be necessary to avoid vascular catastrophe and islet death. It is important to remember, however, that genes are often pleotropic and that genes known to be important for angiogenesis may have a different function in the pancreatic islet endothelial cells. There is some support in the literature for the feasibility of ongoing tissue repair in the islets of the adult human pancreas. In a recent report, Smeets et al. found an increase in M2 macrophages, microvascular density, and islet cell proliferation in the pancreases of human organ donors with prolonged intensive care unit stay^16^. This expands further on an earlier report of increased β-cell proliferation in a subset of organ donors^26^. Thus, there is previous immunohistochemical evidence of ongoing tissue repair and cellular proliferation in human islets, and our data provide further evidence for this at the transcriptome level.

Although we found ample differences between islet- and exocrine tissue endothelial cells, we did not identify any differences between normoglycemic donors and donors with IGM at gene or gene set level. This is a surprising result, considering earlier findings of morphological differences between normoglycemic and diabetic subjects in both human and rodent studies. Previous work using RNA-Seq has identified differences in the expression of several genes in cubital vein endothelial cells derived from non-diabetic patients and patients with type 2 diabetes^27^. However, the study by Beckman et al. involved macrovascular endothelial cells, which are not directly comparable to MVEC. One interpretation that reconciles our data with previous studies would be that the reported differences seen between normoglycemia and dysglycemia reflect vasodilatory or vasoconstrictory processes, which occurs in some rodent models^24,28–30^. Thus, the reported differences in diabetes could reflect alterations at another level of the microvascular system than the endothelial cells. There was, however, a heterogeneity in clinical characteristics among the subjects in our study, such as differences in HbA1c, treatment regime, and presumably type 2 diabetes duration. It is possible that this heterogeneity could mask subtle or conditional differences between microvascular endothelial cells from normoglycemic donors and donors with IGM.

Acinar cells secrete digestive enzymes vital for proper gastrointestinal function. They have high levels of protein synthesis and, during fasting, a correspondingly high protein catabolism^31^. In our exocrine MVEC samples, we found multiple enriched gene sets that relate to biosynthesis and biomolecule catabolism. These findings could reflect an increased turnover of proteins also in these cells. No statistically significant enrichment was found in gene sets related to autophagy or endoplasmic reticulum (ER) stress, albeit there was a tendency towards enrichment in sets related to ER stress.

A murine endothelial cell transcriptome atlas, containing 78 endothelial cell subclusters using single cell RNA-seq sets from 11 different murine tissues^2^, identified marker genes for angiogenesis, interferon signaling and proliferation, as well as tissue specific markers for capillary endothelial cells. Corresponding angiogenesis markers were found to be enriched in our islet samples while corresponding proliferation markers were enriched in our exocrine samples. Our findings may indicate some similarity between endothelial cells from human pancreatic islets and murine kidney, small intestine, and colon.

Our study of the human endothelial cell transcriptome has some limitations. Primarily, the number of organ donors is relatively small. This is inherent to the complicated procedure of obtaining human islets within less than one hour after isolation. However, this is of utmost importance since a period of islet culture could potentially influence the endothelial cell transcriptome. Further, human organ donors with type 2 diabetes are rare and not commonly available for islet research, adding to this limitation in the comparisons between normoglycemia and IGM. Organ donors are subjected to intensive care and critical illness before islet isolation, which may have different effects across tissues and individual donors. It is also possible that the putative differences we have found are affected by preferential acquisition of different endothelial cell subsets in different tissues. For example, younger endothelial cells may be more likely to survive the isolation and sorting process in islet samples. Losses are common in single-cell dissociation and sorting, with a recent study of the human pancreas recovering 4% of the estimated total number of β-cells^32^. Thus, our and other bulk RNA-seq studies require an assumption that cells are not lost in a biased fashion. Another important limitation of our study is the lack of confirmatory results on the protein level supporting the reported transcriptomic findings. However, based on the findings reported in the present communication we will be entitled to obtain a larger number of human islets, allowing proper characterization of the endothelial cell proteome.

To summarize, we present transcriptional profiles from an important cellular component of the human pancreatic islet. Our data demonstrates that islet capillaries differ markedly from their exocrine counterparts, with an enrichment in gene sets related to vascular development and extracellular matrix remodeling. The most parsimonious interpretation would be an ongoing remodulation of the islet vasculature even in the adult human pancreas.

## Methods

### Organ donors and Islet isolation

Transplant-grade human pancreata were collected from brain dead multi organ donors through the Nordic Network for Islet Transplantation. Tissue from 8 donors with normoglycemia (defined as HbA1c < 42 mmol/mol and no diagnosis of diabetes) and 5 donors with impaired glucose tolerance (IGM—defined as diagnosis of type 2 diabetes and treatment for type 2 diabetes, or HbA1c > 42 mmol/mol) was included in the study. Available islet and organ donor data is presented in Table 1. Islets and exocrine tissue were isolated at the islet isolation facility (Rudbecklaboratoriet, Uppsala) using a previously described method^33^. Single cell dissociation was initiated immediately after completion of islet isolation.

**Table 1:** Organ donor data. CIT, Cold Ischemia Time. IA2A, insulinoma-associated protein 2 autoantibody.

Donor	Age	Sex	BMI	HbA1C (mmol/mol; %)	CIT (h:m)	Islet purity	IA2A, GADA status	Notes	Insulin release (Dynamic index)	Cause of death^a^
Normo1	75	F	31.1	35; 5.4	13:05	99%	Negative		2.3	ICH
Normo2	70	F	40.8	42; 6.0	4:39	92%	Negative		1.6	CVL
Normo3	39	M	26.6	34; 5.3	14:34	78%	n/a		18.0	ICH
Normo4	62	M	22.5	29; 4.8	17:35	91%	n/a		6.4	SDH
Normo5	68	F	26.3	40; 5.8	23:51	87%	Negative		19.3	CA
Normo6	49	M	30.9	39; 5.7	17:57	86%	Negative		n/a	CA
Normo7	73	F	21.7	35; 5.4	19:11	87%	Negative		8.9	TBI
Normo8	67	M	26.3	33; 5.2	18:50	100%	Negative	Excluded from some analyses ^b^	1.4	TBI
IGM1	58	M	30.9	48; 6.5	11:48	83%	n/a		n/a	CI
IGM2	78	F	22.9	69; 8.5	18:50	92%	Negative	Type 2 diabetes diagnosis, Metformin treatment	1.2	ICH
IGM3	63	M	27.8	38; 5.6	9:08	92%	Negative	Type 2 diabetes diagnosis, Metformin treatment	7.0	SAH
IGM4	77	M	26.3	40; 5.8	19:24	98%	Negative	Type 2 diabetes diagnosis, Trajenta treatment	4.1	CA
IGM5	55	M	30.5	46; 6.4	15:56	91%	Negative		6.4	ICH, SAH
Normo mean; IGM mean	63; 66	-	28.3; 27.7	36; 48/5.5; 6.6	16:13; 15:01	90%; 91%	–		8.3; 4.7	
Normo median; IGM median	68; 63	-	26.5; 27.8	35; 46/5.4; 6.4	17:46; 15:56	89%; 92%	–		6.4; 5.3	

*GAD* glutamic acid decarboxylase autoantibody,* ICH* intracerebral hemorrhage,* CVL* cerebrovascular lesion,* SDH* subdural hematoma,* CA* cerebral anoxia following cardiac arrest,* TBI* traumatic brain injury,* CI* cerebral infarction,* SAH* subarachnoid hemorrhage.

^a^As entered on the organ donor form.

^b^Islet sample excluded from all analyses due to low quality reads in islet EC library. Exocrine sample used in PCA, heatmap, and comparisons between donors with normoglycemia and donors with IGM.

### Ethics statement

Consent for organ donation (for clinical transplantation and for use in research) was obtained via online database (https://www.socialstyrelsen.se/en/apply-and-register/join-the-swedish-national-donor-register/) or verbally from the deceased's next of kin by the attending physician and documented in the medical records of the deceased in accordance with Swedish law and as approved by the Regional Ethics Committee in Uppsala (Dnr 2009/371/2). The present study protocol was approved by the Regional Ethics Committee in Uppsala (Dnr 2015/444). All experiments were carried out in accordance with applicable regulations and ethical guidelines. No organs were procured from prisoners.

### Single-Cell dissociation and fluorescence-activated cell sorting of endothelial cells

Reagent details are presented in Table 2. From each donated pancreas, 10,000 IEQ were collected from an endocrine tissue isolate (endocrine content of 78–100% based on automated microscopic evaluation after Dithizone staining) and a similar amount of tissue was collected from an exocrine tissue isolate from the same donor (endocrine content of 0–4%). Single cell suspensions were generated through incubation in Accutase for 20 min at room temperature.

**Table 2:** Reagent table.

Reagent	Vendor	Catalog number	Notes
Accutase	Thermo Fisher	A1110501	
CD31 BB515 conjugated antibody, clone WM59	BD Biosciences	564630	RRID:AB_2738872
CD34 BV421 conjugated antibody, clone 581	BD Biosciences	562577	RRID:AB_2687922
CD45 APC-H7 conjugated antibody, clone 2D1	BD Biosciences	560,178	RRID:AB_1645479
MACSQuant Tyto Running Buffer	Miltenyi Biotec	130-107-206	
Buffer RLT+	Qiagen	1,053,393	Included in the RNeasy Plus Mini-Kit
RNeasy Plus Mini-Kit	Qiagen	74134	
SuperScript VILO cDNA Synthesis Kit	Thermo Fisher	11754050	
QuantiTect Primer Assays: INS, GCG, AMY2A, PECAM1, VWF, GAPDH, RRN18S	Qiagen	QT01531040, QT00091756, QT01680595, QT00081172, QT00051975, QT01192646, QT00199367	
Ion AmpliSeq Transcriptome Human Gene Expression Kit	Thermo Fisher	A26325	

The disassociated cells were then stained for CD31-BB515, CD34-BV421, and CD45-APC-H7 for 30 min at 4 °C, washed and resuspended in a sorting buffer (Tyto running buffer). Small aliquots of the antibody-stained cells were collected for later use in flow cytometric and RT-qPCR analysis of bulk samples. Cell suspensions were passed through a cell strainer before sorting. Cells double positive for CD31 and CD34 and negative for CD45 (CD31^+^CD34^+^CD45^−^) were considered endothelial and sorted at 8 °C using a microchip-based fluorescence activated cell sorter (MacsQuant Tyto, Miltenyi) which allows for sterile, low pressure sorting of cells. Purple laser (CD34-BV421) was set as trigger channel and blue laser (CD31-BB515) as speed channel. Aliquots were collected from the sorted cell samples and the purity of the sorted cell population was analyzed on a BD FACSVerse instrument. The remaining cells were lysed in buffer RLT+ and stored at − 70 °C prior to RNA extraction.

### RNA extraction and RT-qPCR

RNA was extracted from the cells lysed in buffer RLT+ using the RNeasy Plus Mini kit according to the manufacturer’s instructions.

RT-qPCR was performed as a quality control measure of successful cell-sorting, targeting endocrine (*INS, GCG*), exocrine (*AMY2A*) and endothelial (*PECAM*, *VWF*) genes. The cDNA used in RT-qPCR was synthesized using the SuperScript VILO cDNA Synthesis Kit. Reactions were run for 40 cycles on a StepOne Plus instrument with settings following the manufacturer’s recommendations. Reactions were run in duplicate and specificity was confirmed with melt curve analysis of the amplified products. Relative expression levels were calculated using the 2^ΔCT^ method, with *GAPDH* and *18S* as reference genes. In one exocrine MVEC sample, relative expression was calculated using *18S* only due to multiple temperature peaks in the *GAPDH* duplicates. Melt curves were not available for one donor due to hardware failure. In another donor, cDNA was not available from bulk exocrine material, which was thus substituted by exocrine flow-through material. These aberrations did not noticeably affect the summarized data.

### Library preparation and transcriptome sequencing

Libraries were prepared for each sample with the Ion AmpliSeq Transcriptome Human Gene Expression Kit. Sequencing of sample pools was then done using the Ion 550 Kit and the S5 XL (IonS5XL, Thermo Fisher) instrument with a 550 chip in 3 separate runs. The primer pool hg19_AmpliSeq_Transcriptome_21K_v1, containing primers for 20,813 amplicons, was used.

Average mean read length for each run was 112, 112 and 113 bp respectively. The produced reads were aligned to hg19 AmpliSeq Transcriptome ERCC v1. The mapping rates were 97%, 96% and 96%.

One sample (the islet EC sample of donor Normo8) produced low quality reads. This sample was thus excluded from data analysis. 13 other samples from pancreatic arteries were sequenced alongside the MVEC samples (possibly affecting the average read lengths and mapping rates of the three runs), but were not included in any data analyses.

### Data normalization, exploration and differential gene expression testing

The raw counts were analyzed using the edgeR^34,35^ package (v3.28.1) in R (v.3.5.3). The filterByExpr function was used to remove any genes that did not have the equivalent of at least 10 CPM (counts per million) in at least 5 samples (the size of the smallest experimental group). Raw counts were normalized using TMM (trimmed mean of M) values^36,37^. TMM adjusted and log-normalized CPM were used for principal component analysis using the R-package PCAtools v.1.2.04^38^. A heatmap with hierarchical clustering was constructed for the 1000 genes with the highest variance using the pheatmap package in R and the calculated z- scores of the logCPM- TMM normalized counts.

Differential gene expressions were assessed for tissue origin and glycemic status using the edgeR functions glmQLFit and glmTreat (quasi-likelihood, QL). Genes with a fold change of >  ± 1.2 and a FDR-value < 0.05 were considered differentially expressed at a significant level.

24 samples from 12 donors were used for differential gene expression analysis using a paired generalized linear model. The exocrine sample from Normo8 was excluded as it lacked a paired islet sample.

### Gene set enrichment analysis

Gene set enrichment analysis (GSEA)^39,40^ was performed with the GSEA software v4.0.3 (Broad Institute, USA), using a gene list ranked with the negative log of the p-value multiplied by the sign of the fold change for the TREAT-analysis between islet and exocrine samples as the ranking metric. 1000 gene permutations and a weighted gene enrichment statistic was used for the analysis. Gene sets containing between 15 and 500 genes from GO^41,42^ biological processes (ftp.broadinstitute.org://pub/gsea/gene_sets/c5.bp.v6.2.symbols.gmt). To test for differences between donors with normoglycemia and donors with IGM, TMM and CPM normalized counts were used and 1000 phenotype permutations were performed.

Gene sets with an FDR q-value < 0.25 and a nominal p-value < 0.01 were considered significantly enriched. Leading edge analysis was performed on gene sets with FDR < 0.05.

Visualization, clustering and annotation of the GSEA result was performed using the Enrichment map suite^43^ within Cytoscape 3.7.2 software^43,44^. A nominal p-value cutoff of 0.005 and a FDR-cutoff of 0.01 was used with a Jaccard + Overlap combined model. Gene set annotations were manually curated.

### Deconvolution analysis

To estimate the proportions of cell types in the samples, raw counts were deconvoluted through MuSic (Multi-Subject Single Cell Deconvolution) using the R-package MuSic (v.0.0.1)^45^ and the single-cell RNA-seq dataset E-MTAB-5061^17^.

### Comparison with murine endothelial cell atlas

Single-cell RNA-Seq data sets from a murine endothelial cell atlas^2^ were converted from MGI to HGNC using the R-package biomaRt^46^. 14 Gene sets corresponding to 11 tissues of origin (heart, brain, liver, extensor digitorum longus, kidney, small intestine, colon, lung, soleus, spleen, testis) for capillary endothelial cells and 3 different biological processes (angiogenic, proliferation, interferon) were constructed from the genes in each set that overlaps with the genes remaining in y after the filtering step. Pre-ranked GSEA was then used to allow comparison with our data sets.

### Data visualization

Data was visualized using GraphPad Prism v7.02, the R package ggplot2 (v.3.3.0), Inkscape(v.1.0) and FlowJo v10.7.1.

### Statistics

Differential gene expression analysis: Differential gene expression was assessed using a paired generalized linear model and a quasi- likelihood function test that tests for a fold change of >  ± 1.2. FDR- values were calculated using the Benjamini–Hochberg method as applied in edgeR. Genes with a FDR-value < 0.05 were considered to differentially expressed at a significant level.

GSEA: A ranked gene list was created using the negative log of the p-value multiplied by the sign of the fold change for the TREAT-analysis between islet and exocrine samples as the ranking metric. 1000 gene permutations and a weighted gene enrichment statistic was used for the analysis. Gene sets with an FDR q-value < 0.25 and a nominal p-value < 0.01 were considered significantly enriched. Leading edge analysis was performed on gene sets with FDR < 0.05. For clustering and visualization, a nominal p-value cutoff of 0.005 and a FDR-cutoff of 0.01 was used with a Jaccard + Overlap combined model.

## Data Availability

Raw counts and TMM-CPM-log2-normalized counts are available on GEO (GSE157546).
